# A Tetraploid Intermediate Precedes Aneuploid Formation in Yeasts Exposed to Fluconazole

**DOI:** 10.1371/journal.pbio.1001815

**Published:** 2014-03-18

**Authors:** Benjamin D. Harrison, Jordan Hashemi, Maayan Bibi, Rebecca Pulver, Danny Bavli, Yaakov Nahmias, Melanie Wellington, Guillermo Sapiro, Judith Berman

**Affiliations:** 1 Department of Genetics, Cell, and Developmental Biology, University of Minnesota–Twin Cities, Minneapolis, Minnesota, United States of America; 2 Department of Electrical and Computer Engineering, Duke University, Durham, North Carolina, United States of America; 3 Department of Molecular Microbiology and Biotechnology, Tel Aviv University, Tel Aviv, Israel; 4 Alexander Grass Center for Bioengineering, The Hebrew University of Jerusalem, Jerusalem, Israel; 5 Department of Pediatrics, University of Rochester Medical Center, Rochester, New York, United States of America; National Cancer Institute, United States of America

## Abstract

*Candida albicans*, the most prevalent human fungal pathogen, is generally diploid. However, 50% of isolates that are resistant to fluconazole (FLC), the most widely used antifungal, are aneuploid and some aneuploidies can confer FLC resistance. To ask if FLC exposure causes or only selects for aneuploidy, we analyzed diploid strains during exposure to FLC using flow cytometry and epifluorescence microscopy. FLC exposure caused a consistent deviation from normal cell cycle regulation: nuclear and spindle cycles initiated prior to bud emergence, leading to “trimeras,” three connected cells composed of a mother, daughter, and granddaughter bud. Initially binucleate, trimeras underwent coordinated nuclear division yielding four daughter nuclei, two of which underwent mitotic collapse to form a tetraploid cell with extra spindle components. In subsequent cell cycles, the abnormal number of spindles resulted in unequal DNA segregation and viable aneuploid progeny. The process of aneuploid formation in *C. albicans* is highly reminiscent of early stages in human tumorigenesis in that aneuploidy arises through a tetraploid intermediate and subsequent unequal DNA segregation driven by multiple spindles coupled with a subsequent selective advantage conferred by at least some aneuploidies during growth under stress. Finally, trimera formation was detected in response to other azole antifungals, in related *Candida* species, and in an *in vivo* model for Candida infection, suggesting that aneuploids arise due to azole treatment of several pathogenic yeasts and that this can occur during the infection process.

## Abstract 2

When exposed to the antifungal drug fluconazole, *Candida albicans* undergoes abnormal growth, forming three-lobed “trimeras.” These aneuploid trimeras turn out genetically variable progeny with varying numbers of chromosomes, increasing the odds of creating a drug-resistant strain.

## Author Summary

Fungal infections are a particularly challenging problem in medicine due to the small number of effective antifungal drugs available. Fluconazole, the most commonly prescribed antifungal, prevents cells from growing but does not kill them, giving the fungal population a window of opportunity to become drug resistant. *Candida albicans* is the most prevalent fungal pathogen, and many fluconazole-resistant strains of this microbe have been isolated in the clinic. Fluconazole-resistant isolates often contain an abnormal number of chromosomes (a state called aneuploidy), and the additional copies of drug resistance genes on those chromosomes enable the cells to circumvent the drug. How *Candida* cells acquire abnormal chromosome numbers is a very important medical question—is aneuploidy merely passively selected for, or is it actively induced by the drug treatment? In this study, we found that fluconazole and other related azole antifungals induce abnormal cell cycle progression in which mother and daughter cells fail to separate after chromosome segregation. Following a further growth cycle, these cells form an unusual cell type that we have termed “trimeras”—three-lobed cells with two nuclei. The aberrant chromosome segregation dynamics in trimeras produce progeny with double the normal number of chromosomes. Unequal chromosome segregation in these progeny leads to an increase in the prevalence of aneuploidy in the population. We postulate that the increase in aneuploidy greatly increases the odds of developing drug resistance.

## Introduction

Fungal pathogens have a profound effect on human health, causing millions of deaths worldwide [1]. *Candida albicans* is among the most prevalent fungal human pathogens [1] and was long thought to be an obligate diploid (2N DNA content). Although true meiotic divisions have not been detected in *C. albicans*, tetraploids (4N DNA content) form *in vitro*, via mating between diploids [2]–[4], and undergo “concerted chromosome loss” (CChrL), a nonmeiotic reduction in chromosome number [5]. The mechanism driving CChrL is unclear, but it yields near-diploid progeny that often carry 1–3 aneuploid chromosomes [6]. Haploid *C. albicans* recently were discovered as well, apparently forming via a nonmeiotic CChrL process similar to that seen in the tetraploid-to-diploid transition [7]. Haploids can reestablish a diploid state via mating or via autodiploidization, a poorly understood process presumed to occur via either endoreduplication or a complete failure of mitosis. The existence of semistable, nondiploid *C. albicans* cell types highlights the flexibility of the *C. albicans* genome. It also raises important questions about how nondiploid isolates, particularly aneuploids, arise and are maintained especially in light of the fitness cost associated with aneuploidy in the model yeast *Saccharomyces cerevisiae* under optimal growth conditions [8]–[11].

Fluconazole (FLC), the most commonly prescribed antifungal drug, is a triazole that specifically targets Erg11p, a lanosterol 14(α)-demethylase. Inhibition of Erg11 interferes with ergosterol biosynthesis and membrane integrity [12]. FLC is fungistatic, rather than fungicidal, thereby providing opportunities for fungal cells to develop FLC resistance (FLC^R^). Importantly, many (∼50%) FLC^R^ isolates are aneuploid and at least one specific aneuploidy, an isochromosome of the left arm of chromosome 5 (i5L), clearly confers FLC^R^ by providing extra copies of two genes important for resistance [13],[14]. Although selection may influence the types of aneuploidies that appear in FLC^R^ isolates, the mechanisms by which aneuploid FLC^R^ isolates arise at such high frequency are not known.

*C. albicans* grows with three different morphotypes; yeast, pseuodohyphae, and true hyphae [15] and the *C. albicans* yeast cell cycle resembles the well-characterized cell cycle of *S. cerevisiae*: bud growth, DNA replication, and mitotic spindle assembly are coordinated during each cell cycle. Bud emergence, DNA replication, and spindle pole body (SPB) duplication initiate at the same time point, termed START, in late G1 phase. Chromosome segregation ensues after DNA replication is completed, buds have grown large enough to receive a nucleus, and the bipolar spindle is ready to execute the metaphase-to-anaphase transition so that both the mother and daughter cell receive a complete genome. In contrast to the yeast cell cycle, *C. albicans* hyphae exhibit more flexibility in cell cycle coordination: evagination of a germ tube (new hypha) precedes the initiation of other START events [16]. Additionally, germ tube formation can be induced in cells that are not in G1 [16]. Thus, the relationship between the nuclear/spindle cycle and the cell growth cycle is regulated differently in *C. albicans* hyphal cells.

In this study, we analyzed the cell cycle events that occur in *C. albicans* yeast cells during exposure to FLC. We found that dramatic changes in DNA content and cell morphology arose through an ordered series of aberrant cell cycle events. First, cell cycle milestones such as the initiation of replication, SPB duplication, and spindle elongation preceded bud emergence and bud growth. Second, cytokinesis failure yielded “trimeras,” binucleate cells with three connected compartments/buds, which were viable and able to form colonies in both the presence and the absence of FLC. Third, trimeras often produced tetraploid progeny cells with 4C nuclei and two spindles via mitotic collapse. Fourth, tetraploid nuclei with multiple spindles often segregated unequally, producing cells with a wide range of DNA content per nucleus, which we interpret as aneuploids. Finally, trimeras yielded nondiploid progeny at high frequency, indicating that the cells with altered ploidy do not incur a severe fitness cost. Furthermore, we propose that the ability to produce aneuploids at high frequency yields a highly diverse population upon which the selective forces of drug exposure can act.

## Results

### FLC Exposure Results in a Subpopulation of Live Cells with Increased DNA Content and Cell Size

**Figure 1:**
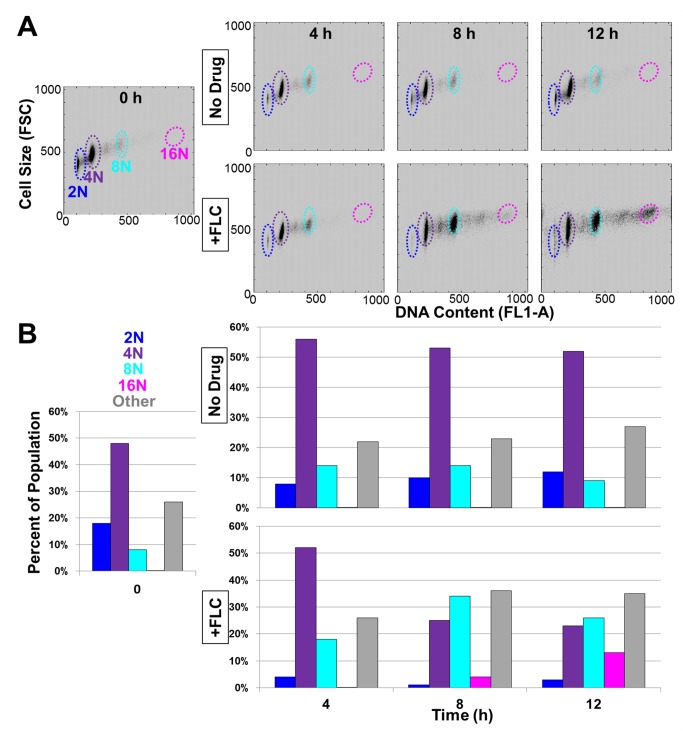
Cell size correlates with DNA content. (A) Flow cytometry data plotted as cell size (FSC) versus DNA content (FL1-A) for cultures grown in the absence (top row) or presence (bottom row) of FLC for times indicated. (B) Bar graphs represent percent of cells that fell into colored regions defined in scatterplots above as determined using Gaussian fitting (see Materials and Methods).

Fifty percent of FLC^R^ *C. albicans* isolates are aneuploid [8],[13]. Although some aneuploidies provide a selective advantage in the presence of FLC [13], it is unknown if the rate of aneuploid formation is increased or if rare aneuploid cells within the population are actively selected in response to FLC exposure. To address this question, we measured the DNA content of log-phase yeast cell populations as a function of time of exposure to FLC (10 µg/ml) using flow cytometry (Figure 1 and Figure S1). Diploid cells not exposed to FLC (“no drug”) consistently appeared in two peaks corresponding to the G1-phase (2N DNA content) and G2-phase (4N DNA content) of the cell cycle, respectively (Figure S1, top two rows). In contrast, after 8 h of FLC exposure, a substantial 8N peak (34% of the population) became evident. After 12 h of FLC exposure, 8N (26%) and 16N (13%) peaks made up over one third of the population, suggesting that a subpopulation of cells had 4N and/or 8N DNA content. No additional features were detected in flow cytometry profiles after 16 and 20 h, suggesting that maximum variability in ploidy levels occurred at 12 h of FLC exposure (Figure S1, bottom two rows).

As ploidy is proportional to cell size [7],[17], we asked if FLC-exposed cells with increased ploidy were also larger in size by analyzing the forward scatter within the flow cytometry output (Figure 1A). Notably, FSC correlated with DNA content (Pearson correlation coefficient of 0.68, Spearman correlation coefficient of 0.76), indicating that cells containing more DNA were also larger. Thus, after 8 h of FLC exposure, roughly one third of the population (38%) was large cells with increased DNA content.

### Abnormally Large Cells Are Multinucleate and Viable

**Figure 2:**
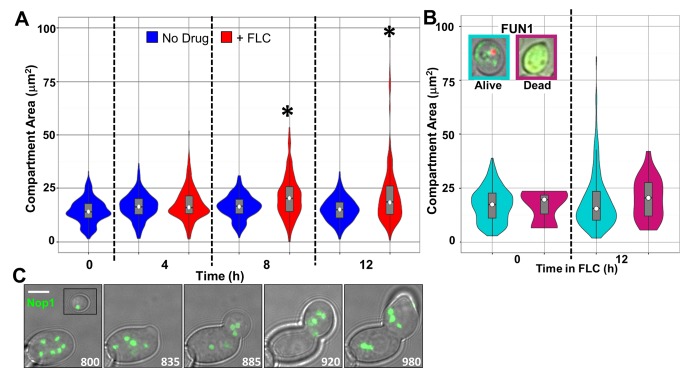
Abnormally large cells are viable. (A) Violin plots (area indicates frequency along the *y*-axis) overlaid with box plots (gray) showing the distribution of cell sizes (see Materials and Methods). “No drug” control cultures (blue) show no significant changes in cell size distribution over time. FLC-exposed cultures (red) show a significant cell size increase at 8 and 12 h of FLC exposure (two-tailed *t* test, *p* value <0.05). (B) Violin plots, as above, showing size distribution of metabolically active (cyan, alive) and inactive (magenta, dead) cells as determined by FUN1 staining interpreted as shown in the micrograph insets. (C) Abnormally large cells with multiple nucleoli (detected with Nop1-GFP, green) continued to grow, produced new buds, and underwent unusual nucleolar segregation patterns. Inset in first panel is an unbudded cell from a “no drug” control shown on the same size scale. Scale bar, 5 µm.

To directly investigate the relationship between ploidy and cell size, we imaged cells during the course of FLC exposure using differential interference contrast (DIC) microscopy. Upon exposure to FLC, average cell size increased from 15.92 µm^2^ to 20.39 µm^2^ and 18.50 µm^2^, at 4, 8, and 12 h, respectively, whereas no significant changes were observed for cells not exposed to FLC (Figure 2A). Average cell size was significantly larger in FLC-exposed cultures when compared to unexposed cultures at 8 and 12 h (*t* test, *p* value <0.05). The larger cells also formed chains of cells, presumably because cytokinesis had failed during multiple rounds of cell duplication. We termed these abnormal cells multimeras, and we describe them in more detail below. Although these abnormal cells could have been a product of cell fusion, we do not think this is likely because when we mixed cells expressing a constitutive GFP marker with cells expressing a constitutive mCherry marker during FLC exposure, no cells expressing both markers were detected (unpublished data). Together, the microscopy and flow cytometry data indicate that major changes to *C. albicans* cell ploidy and morphology occurred after 4 h of FLC exposure.

To investigate the viability of the abnormally large cells, we stained cultures with FUN1, which stains the intravacuolar structures of metabolically active cells red and the cytoplasm of dead cells green (inset, Figure 2B). The majority of the very large cells in FLC-exposed cultures (>2 SD above the mean cell size of “no drug” populations) were metabolically active. Consistent with this, most large multimeras continued to grow and nucleoli within them continued to divide as detected by time-lapse microscopy using the nucleolar protein Nop1 fused to GFP (Figure 2C) [18]. Astonishingly, many large budded multimeras had multiple nucleoli or, at least, had multiple copies of ChrR, which encodes the rDNA and is thus associated with the nucleoli (Figure 2C, Movie S1), thus implying that they may have multiple nuclei. Furthermore, these cells continued to bud and the multiple nuclei sometimes appeared to coalesce or collapse onto one another. Taken together, the flow cytometry, time course, and time-lapse microscopy data indicate that, over time, exposure to FLC promotes development of a subpopulation of very large, multinucleolar cells that appeared to form metabolically active chains and that often exhibit unconventional nucleolar (and presumably nuclear) dynamics.

### Nuclear/Spindle Cycles Initiate Prior to Bud Emergence in the Presence of FLC

To elucidate mechanisms contributing to the ploidy and cell size changes that occurred during FLC exposure (Figure 1), we performed time-lapse microscopy focusing on different times, starting with the 4–8 h time period. We monitored bud growth by DIC microscopy of cell morphology, the nuclear cycle with Nop1-RFP, and the spindle cycle with Tub1-GFP, a microtubule marker. We focused attention on classic cell cycle landmarks: (1) bud emergence, (2) spindle assembly, (3) anaphase onset, (4) nucleolar separation/segregation, and (5) spindle disassembly.

**Figure 3:**
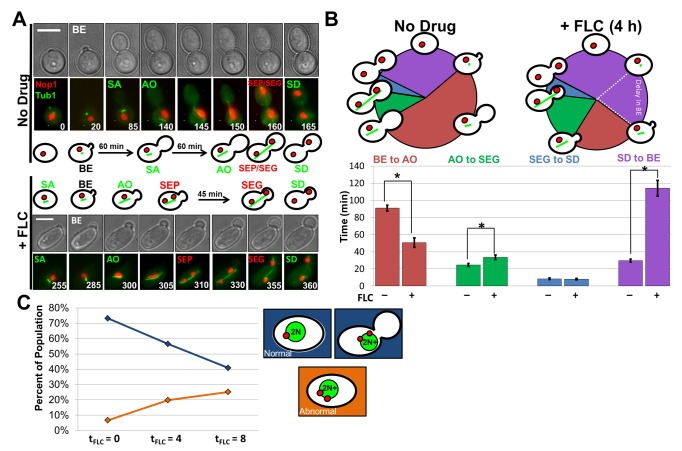
Bud emergence is delayed after 4 h of FLC exposure. (A) Individual frames from time-lapse images of Nop1-RFP/Tub1-GFP–expressing cells in the absence (top two rows) and presence (4 h, bottom two rows) of FLC. BE, bud emergence; SA, spindle assembly; AO, anaphase onset; SEP, sister separation; SEG, sister segregation across bud neck; SD, spindle disassembly. Illustrations in rows 3 and 4 show relative timing of events. Numbers in fluorescent images denote time (min) of FLC exposure. Scale bars, 5 µm. (B) Cartoon illustrating relative timing of cell cycle events in the absence (left) and presence (right) of FLC. Lower left panel, average time between bud emergence (BE, purple) and anaphase onset (AO, gray) events. Bar graph, average time between BE and AO (red), AO and SEG (green), SEG and SD (blue), and SD and BE (purple). Error bars are 1 standard deviation from mean based on *n* = 25 cells observed on 2 d. Asterisks denote statistically significant differences (*t* test, *p* value <0.05). (C) Proportion of cells with normal pre-START and pre-anaphase phenotypes (blue) and aberrant START (unbudded cells with two SPBs, yellow) phenotypes. Cells not represented here were in mitosis. “No drug” control cultures showed no significant deviation from t = 0 numbers (unpublished data).

Stereotypical cell cycle dynamics were observed in the “no drug” condition (Figure 3A, Movie S2). Unbudded cells contained a single nucleolar mass. Coincident with bud emergence, a single Tub1-GFP focus appeared, indicative of the initiation of spindle assembly. Assembly of a short spindle, visualized as a bar of GFP fluorescence, was completed approximately 1 h later. Anaphase onset detected as elongation of the mitotic spindle, occurred after 2 h. Within 10 min of anaphase onset, the spindle elongated and traversed the bud neck, followed rapidly (within 10 min) by sister nucleolar separation and segregation (Figure 3A, second row, 160 min). Spindle disassembly occurred 10 min after nucleolar segregation. An average cell cycle lasted 153.6±12.0 min from bud emergence to bud emergence or 151.2±30.6 min from anaphase onset to anaphase onset (Figure 3B). The similarity between these two measurements implies that the bud growth cycle and the nuclear/spindle cycles are coordinated.

After 4 h of FLC exposure, cultures contained subpopulations of cells that began to display atypical cell cycle dynamics with four new features. Unbudded cells contained a short spindle, detected as a bar of Tub1-GFP fluorescence (Figure 3A, bottom two rows; Movie S3), indicating that SPB duplication preceded bud evagination. Anaphase onset (spindle elongation) occurred in cells with buds significantly smaller than cells at the same stage in the “no drug” condition (bud size 41.0%±11% versus 74.2%±9% the size of the mother, respectively; *t* test, *p* value <0.05), indicating that nuclear division preceded the formation of a bud large enough to contain the daughter nucleus. Third, spindle elongation occurred within the mother cell and sister nucleoli separated despite a failure of the spindle to traverse the bud neck. Instead, the mitotic spindle persisted in an elongated state for 30 to 50 min, whereas in “no drug” control cells this process took only 25 min or less. Following nucleolar segregation into a bud, spindle disassembly occurred as rapidly as it was observed in “no drug” cells (within 10 min), suggesting that normal spindle disassembly was triggered once the spindle and a daughter nucleus crossed the bud neck. In summary, after 4 h of FLC exposure, cells exhibited (1) spindle assembly prior to bud emergence, (2) anaphase onset in cells with small buds, and (3) sister nucleolar separation prior to segregation of the nucleus across the bud neck. We suggest that this order of events is due to delayed bud emergence/growth and a failure to coordinate bud growth with the spindle assembly and DNA replication/segregation. Consistent with this, the time between bud emergence and anaphase onset was relatively short (e.g., Figure 3A, average times; Figure 3B), while the time between spindle elongation initiation and nucleolar segregation (Figure 3A, bottom row, AO and Seg, respectively; Figure 3B, average times) was relatively long, compared to “no drug” cells. The time between segregation and spindle disassembly was not altered in these cells (Figure 3B); however, the time between spindle disassembly and bud emergence in the next cell cycle was drastically increased in FLC-exposed cells compared to no drug controls (Figure 3B).These alterations in the cell cycle are consistent with an altered relationship between the bud growth cycle and the nuclear and spindle cycles.

To quantitate the differences in cell cycle timing between “no drug” and FLC-exposed cells, we quantified the amount of time that cells spent in different cell cycle stages using a nuclear marker (histone H4 fused to GFP (Hhf1-GFP)), rather than the Nop1-GFP nucleolar marker. SPB duplication was followed using Tub4, the presumed γ-tubulin subunit of SPBs, fused to mCherry (Tub4-mCherry) in the same cells. The amount of DNA per cell was determined by measuring the fluorescence intensity of Hhf1-GFP (manuscript in review). In FLC-exposed cultures (4–8 h), the proportion of the population in stereotypical G1 (unbudded, 1 SPB, 2N DNA content) and S/G2 (budded, 1 nucleus, 2 SPBs, >2N content) decreased (73%, 57%, and 41% at 0, 4, and 8 h, respectively; Figure 3C), while the proportion of unbudded cells with 2 SPBs and S/G2 DNA content (between 2N and 4N) increased (7%, 20%, and 25% at 0, 4, and 8 h, respectively; Figure 3C). This indicates that the coordination of START events (bud emergence, DNA replication initiation, and SPB duplication) [19]–[21] was altered, with bud emergence delayed relative to DNA replication initiation and SPB duplication. Importantly, despite the cell cycle delay, there is no evidence of altered ploidy, as either whole genome ploidy shifts or aneuploidy at these early times after FLC exposure (Figure 1). These data are consistent with the time-lapse data of cells exposed to FLC for 4 h in that DNA replication, in addition to nucleolar cycles, proceed while bud emergence and bud growth lag behind.

### Initial Formation of Cells with Unusual Morphologies: Trimeras

**Figure 4:**
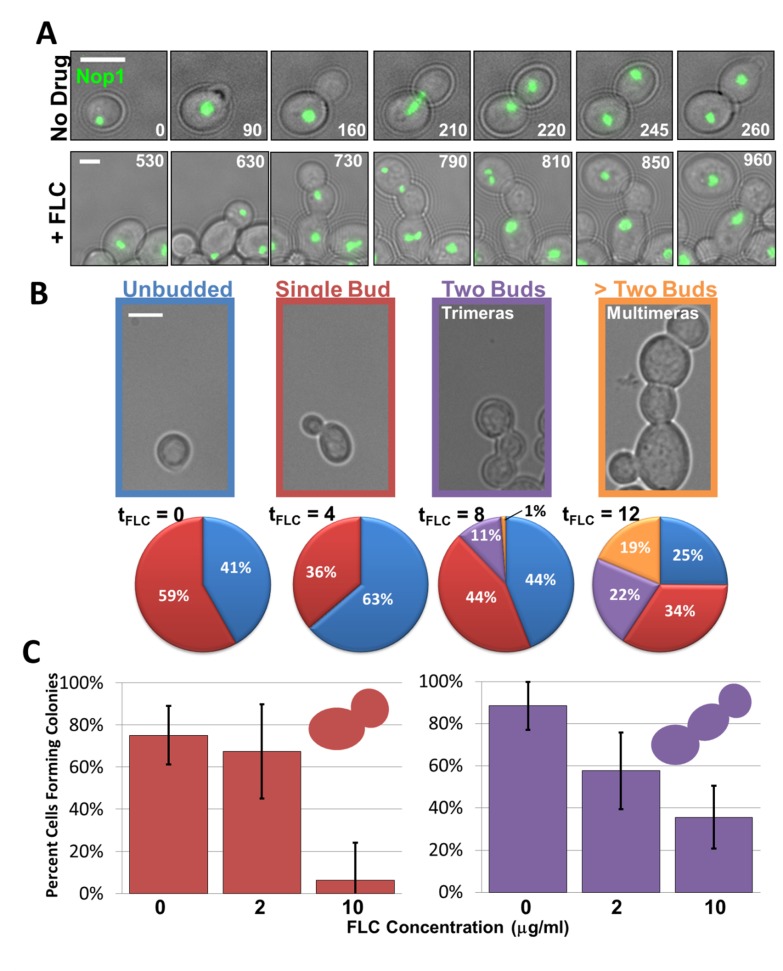
Trimeras become prevalent after 8 h of FLC exposure and remain viable. (A) Time-lapse microscopy of control (top row) and FLC-exposed cells expressing Nop1-GFP. Numbers indicate time (min) of FLC exposure. (B) Relative proportion of cells with indicated morphologies (indicated in color-outlined images) at different times (t, time) of FLC exposure. “No drug” control cultures showed no significant deviation from t = 0 numbers (unpublished data). Colors in pie charts correspond to color of image outline. Scale bars, 5 µm. At least 300 cells from each of two different strains were analyzed for each time point. (C) Colony formation assay following cell micromanipulation. Following 12 h pre-exposure to 10 µg/ml FLC, single budded cells (red) and trimeras (purple) were transferred to plates with indicated FLC concentration. The number of cells analyzed for single budded cells on 0, 2, and 10 µg/ml FLC plates were 52, 53, and 48, respectively. The number of trimeras analyzed on 0, 2, and 10 µg/ml FLC plates were 78, 65, and 48, respectively. Error bars indicate standard error, and statistical significance was determined using a Fisher's exact test.

We used time-lapse microscopy of cells exposed to FLC for 6–8 h to monitor cell morphology (with phase-contrast microscopy) and nucleolar dynamics (with Nop1-GFP) in an effort to uncover the initial cellular defects related to FLC exposure. Strikingly, in FLC-exposed cultures, an unusual “trimera” cell morphology became evident (Figure 4A, bottom row panels; Movie S4). Trimeras were composed of three compartments (mother, daughter, and granddaughter) that shared a single contiguous cytoplasm as subcellular structures were able to pass through the two bud necks (Figure S2). Thus, cytokinesis between the original mother and daughter had failed and the pair of cells produced only a single granddaughter bud (Figure 4A, bottom row, 730 min). Interestingly, even though cytokinesis had failed between mother and daughter, following mitosis of the two nuclei, cytokinesis and cell separation were observed between daughter and granddaughter (Figure 4A, bottom row, 960 min). In time course studies, trimeras appeared at 8 h and became more prevalent after 12 h of FLC exposure (11% and 22% of cells, respectively) (Figure 4B, purple). Similar timing and frequency of trimera formation were found in several strains, including strains without tagged proteins. In contrast, no trimera formation was detected in a FLC^R^ clinical isolate, indicating that susceptibility to FLC is necessary for trimera formation (Figure S3). Taken together, we infer that reduced coordination of the bud growth and nuclear/spindle cell cycles lead to a cascade of events, including abrogation of cytokinesis, that result in the formation of binucleate trimeras.

### Trimeras Are Viable and Can Survive in the Presence of Continued Drug Exposure

Because trimeras became so prevalent in FLC-exposed cultures, we asked if trimeras are viable and able to form colonies. We isolated trimeras by micromanipulation from a culture exposed to FLC for 12 h. Since 99% of “no drug” cells formed colonies after micromanipulation, we assumed that micromanipulation per se did not contribute significantly to cell death (unpublished data). Micromanipulated trimeras formed colonies both in the presence and in the absence of FLC. Trimeras and cells with single buds formed colonies at similar frequencies both on no drug (88% versus 75%, *p* = 0.057) as well as on plates with 2 µg/ml FLC (58% versus 67%, *p* = 0.36) (Figure 4C). Importantly, on a higher FLC concentration (10 µg/ml) trimeras formed significantly more colonies than did single budded cells (36% versus 6%, *p* = 0.0027 calculated per cell, *p* = 0.010 calculated per nucleus; Figure 4C). Thus, trimeras represent a novel intermediate that arises in the presence of FLC and is capable of growing at least as well as “normal” single-budded cells in both the absence and presence of FLC.

### Trimera Progeny Include Diploids, Tetraploids, and Dikaryons

**Figure 5:**
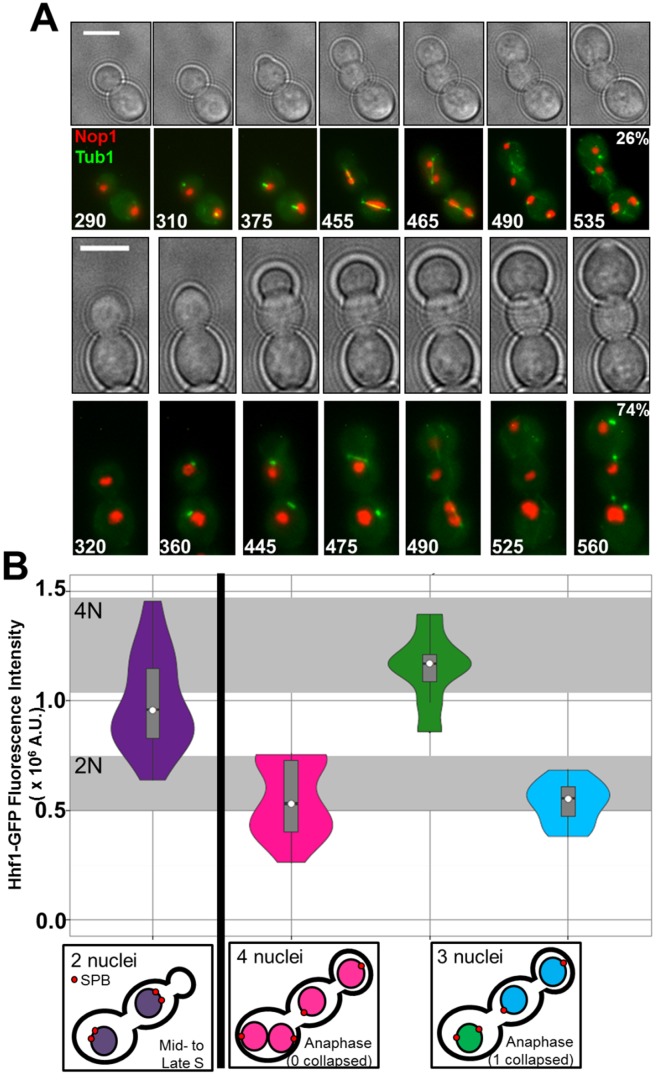
Nuclei within trimeras undergo mitotic collapse to form tetraploids. (A) Time-lapse images of trimera formation and mitotic dynamics. Numbers in fluorescent images denote time in minutes. Also indicated is the percent of trimeras (*n* = 19) that formed four individual nucleoli (top sequence) and that formed three nucleoli (lower sequence) Scale bars, 5 µm. (B) Violin plots of nuclear DNA content (Hhf1-GFP fluorescence) and SPB number (Tub4-mCherry foci). Colors correspond to measurements of individual nuclei within cells as illustrated below plots: trimeras with two nuclei (purple, *n* = 56 nuclei), trimeras with four nuclei (pink, *n* = 24), and trimeras with three nucleoli, one large (green, *n* = 11) and two small (cyan, *n* = 22). Gray regions define average fluorescence intensity of 2N and 4N nuclei ±1 standard deviation based on log-phase “no drug” control cells.

Both nuclei within trimeras underwent replication and mitosis. Binucleate trimeras necessarily underwent unusual mitoses, in which the four daughter nuclei segregated within three cell compartments. Consistent with previous observations, time-lapse microscopy (Figure 5A, Movie S5, and Movie S6) revealed that mother-daughter pairs failed to undergo cytokinesis prior to emergence of the third trimera bud (the granddaughter). During growth of the granddaughter, each nucleolus was associated with a short spindle (Tub1-GFP bar) consistent with both nuclei proceeding through S/G2 despite the presence of only one new bud (Figure 5A, row 2, t = 310 and row 4, t = 360). The two short spindles elongated, usually simultaneously, producing four daughter nucleoli within the three trimera cell compartments. These four daughter nucleoli underwent one of two different nucleolar segregation patterns. In both cases, two of the three cell compartments contained a single nucleolus. In the third cell compartment, the two daughter nucleoli either remained separated to form an apparently dikaryotic cell compartment (in 5/19 trimeras) or they collapsed back together (Figure 5A, row 4, t = 490–525) prior to cytokinesis, generating an apparently tetraploid cell nucleus. This “mitotic collapse” event occurred in at least one of the nuclei within the majority of the trimeras observed (14/19) and either within a single continuous nuclear envelope or with the re-fusion of two separated sister nuclei (Figure S4). Thus, in either case, trimeras yielded one compartment that either had two separate nucleoli or two overlapping nucleoli. Importantly, these compartments also maintained multiple spindles, observed as green foci (Figure 5A, row 2, t = 535 and row 4, t = 560).

We next measured the amount of chromosomal DNA in each nucleus, using the fluorescence intensity of Hhf1-GFP as a proxy. In separate studies, we found that Hhf1-GFP fluorescence intensity is proportional to the amount of chromosomal DNA in nuclei (manuscript in review), consistent with previous studies that used GFP-tagged histone as a proxy for ploidy [22]. The fluorescence intensity corresponding to 2N and 4N nuclei was based on the fluorescence intensity of individual anaphase/telophase nuclei (2N) and late metaphase nuclei (4N) in “no drug” cells. Immediately following emergence of the granddaughter bud, trimeras had two nuclei that each had a median fluorescence intensity of 0.96×10^6^ A.U. (Figure 5B, purple), indicative of 2N–4N DNA content characteristic of S-phase cells. Each nucleus was associated with a single spindle (2 SPBs within 1 µm of each other). In contrast, in postanaphase trimeras that had a large granddaughter bud and four nuclei, each nucleus was associated with a single SPB and had a median fluorescence intensity value of 0.53×10^6^ A.U., indicative of 2N DNA content and characteristic of telophase or G1 cells. Importantly, in postanaphase trimeras that had only three nuclei, two of the nuclei had a single SPB each and a median fluorescence intensity value of 0.55×10^6^ A.U. (G1 cells with 2N DNA content), while the third nucleus was associated with 2 SPBs and had a median fluorescence of 1.17×10^6^ A.U. (4N DNA content). Taken together, these results imply that in early trimeras both 2N nuclei enter S-phase and undergo mitosis in an attempt to create four 2N nuclei. In 75% of the trimeras, one of the two spindles collapsed to form one tetraploid nucleus that maintained two independent SPBs; the remaining 25% of the trimeras contained four independent diploid nuclei, with two sharing a single cell compartment.

Of note, despite the unusual mitotic events that occurred up to this point (8 h of FLC exposure), the nuclear DNA content of all cells in the FLC-exposed cultures appeared to be essentially euploid—either diploid or tetraploid—but not aneuploid (Figure 5B). It also is important to note that trimeras could undergo cytokinesis after an aberrant mitosis, to yield an unbudded tetraploid granddaughter cell (e.g., Figure 4A, 960 min, top left cell). Consistent with this, the proportion of 8N nuclei increased dramatically after 8 h (Figure 1).

### Aneuploids Arise from Mis-Segregation of Tetraploid Nuclei

**Figure 6:**
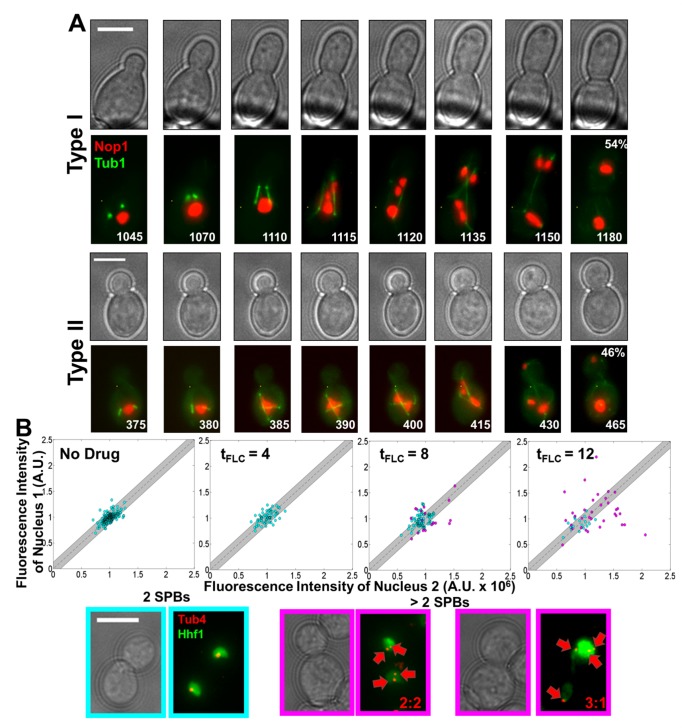
Unequal segregation occurs in nuclei with more than one spindle. (A) Time-lapse microscopy of nuclear segregation patterns in tetraploid/diakaryotic cells. Type I segregation pattern (top two rows, 54% of events), both spindles elongated across the bud neck. Type II (bottom two rows, 46% of events), only one spindle elongated across the bud neck. Numbers denote time (min) of FLC exposure. Scale bars, 5 µm. Total of 13 cells analyzed. (B) Histone H4 (Hhf1)-GFP fluorescence intensity scatter plots. Sister nuclei are plotted relative to each other. Postanaphase cells containing a total of two SPBs (cyan) clustered around 1∶1, indicative of equal segregation (gray region, contains 95% of points from “no drug” cells). Postanaphase cells containing more than two SPBs (magenta) diverged significantly from 1∶1 at 12 h (two-tailed *t* test, *p* value <0.05).

Nuclei containing extra centrosomes (analogous to yeast SPBs) are a hallmark of human cancers and are thought to drive the unequal segregation of chromosomes and the rampant aneuploidy that occurs in many tumorigenic cells [23],[24]. Since trimeras produced cells with 4N DNA content and two spindles, we next followed the segregation of DNA in these individual tetraploids. Tetraploids underwent one of two major nucleolar segregation patterns with similar frequency: during type I segregation, both spindles in the tetraploid cell elongated and traversed the bud neck (7 of 13 divisions) (Movie S7). Alternatively, during type II segregation, both spindles elongated but only one spindle traversed the bud neck (6 of 13 divisions) (Movie S8). In both segregation types, the two spindles initiated elongation simultaneously (type I, Figure 6, row 2, t = 1,110; type II, row 4, t = 380). In type I, both spindles elongated across the bud neck (Figure 6, row 2, t = 1,110–1,120) and remained relatively parallel to one another. In type II, only one of the spindles traversed the bud neck (Figure 6, row 4, t = 385–430). Thus, two spindles could elongate and traverse the bud neck within the same budding cell, but in approximately half of these divisions, both spindles elongated and only one traversed the bud neck.

Based on the assumption that whole chromosome aneuploidies arise through chromosome nondisjunction events and that such events can be detected as unequal segregation of DNA in sister nuclei, we measured the relative amount of DNA in pairs of sister nuclei immediately following their mitotic segregation (Figure 6B). In “no drug” cultures, the 1∶1 ratio of Hhf1-GFP in sister nuclei was used to define “equal” segregation (Figure 6B, 95% of data within grey zone for “no drug” control). No unequal segregation was evident after 4 h of FLC exposure (*p* value >0.05 relative to “no drug” conditions), consistent with flow cytometry and time lapse microscopy results (Figure 1 and Figure S1). Unequal segregation was detected in 8% of cells after 8 h of FLC exposure. Importantly, unequal DNA segregation was associated with cells having more than one SPB per nucleus (83%) (Figure 6B, magenta). In contrast, the vast majority (95%) of anaphase/telophase cells with equal segregation ratios had only one SPB per nucleus (Figure 6B). At 12 h, 29% of cells exhibited unequal segregation ratios and 72% of them had more than one SPB for at least one of the two daughter nuclei. Thus, nuclei with more than two SPBs are much more likely to undergo unequal nuclear segregation. Furthermore, since the majority of trimeras form at least one tetraploid or dikaryotic cell and these 4N cells retain two spindles, this implies that 4N trimera products have a high likelihood of undergoing unequal DNA segregation to produce aneuploids.

### Trimeras Give Rise to Aneuploid Progeny

**Figure 7:**
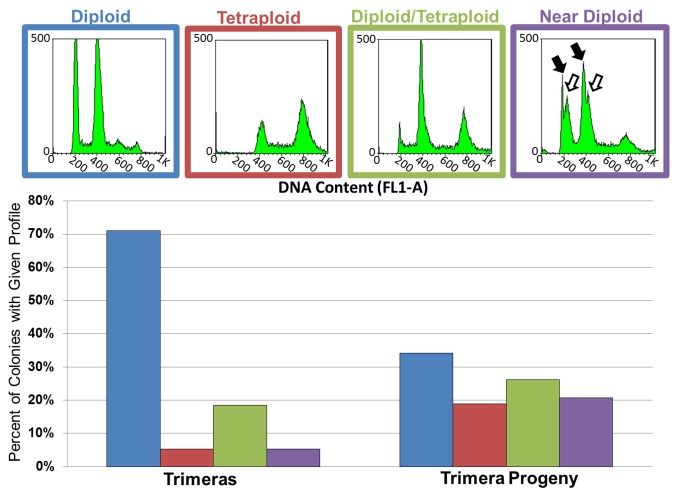
Trimeras give rise to aneuploid progeny. Representative diploid, tetraploid, diploid/tetraploid mixed, and near diploid flow cytometry profiles showing DNA content of populations of cells derived from trimeras (*n* = 40) and their progeny (*n* = 134). Black arrows denote diploid peak pair; white arrows denote near-diploid peak pair. Profile outlines correspond to bars in bar graph showing the percent of trimeras (left) and trimera progeny (right) that exhibited each profile type.

The ploidy alterations due to aberrant mitotic events occurring in trimera and trimera progeny described above were inferred from microscopy of Hhf1-GFP. We next asked if aneuploidy could be detected in trimeras and trimera progeny using flow cytometry. Trimeras that formed following 12 h of FLC exposure were isolated via micromanipulation onto YPAD medium. Progeny cells were micromanipulated away from the parent trimera onto a different area of the same plate. This was done for 1–3 progeny cells per trimera. We then measured the DNA content of the colony derived from the original trimeras (those that remained at their original position) and of the colonies derived from the manipulated trimera progeny. Nondiploid flow cytometry profiles were evident in 29% of trimera-derived colonies and 66% of trimera progeny-derived colonies. Most of these were due to mixed populations of tetraploid and diploid cells (Figure 7). Interestingly, 5% of trimeras and 21% of trimera progeny exhibited near-diploid peak pairs, indicating a subpopulation of aneuploid cells and diploids (empty arrows, Figure 7). These data support the hypothesis that trimeras frequently give rise to viable aneuploid cells within cultures exposed to FLC and that these aneuploidies are retained long enough to detect them during formation of a colony in the absence of drug selection. These data also suggest that the tetraploid and aneuploid states are not completely stable and that nondiploids either return to the diploid state (presumably via chromosome loss) or are out-competed by diploids in the population.

### Non-*albicans* Species Form Trimeras in Response to FLC

Trimera intermediates form aneuploids because of the conundrum of producing four sister nuclei within the three-lobed trimera cell. Thus, trimeras may be a first indication of cells that are likely to become aneuploid. Accordingly, we asked if other pathogenic fungi also produce trimeras. We exposed cultures of *Candida dubliniensis*, *Candida parapsilosis*, *Candida tropicalis*, *Candida lusitaniae*, *Candida glabrata*, and *Saccharomyces cerevisiae* to FLC. Prior to imaging, cells were stained with DAPI in order to visualize nuclear DNA. As expected, no trimeras were detected in any of the “no drug” cultures (Figure 4). In contrast, multinucleate cells resembling trimeras were evident in *C. dubliniensis*, *C. lusitaniae*, *C. parapsilosis*, and *C. tropicalis* (all members of the CUG clade of yeasts that includes *C. albicans*), as well as in *C. glabrata*, a haploid human pathogen more closely related to *S. cerevisiae* (Figure S5). In contrast, trimeras did not appear in *S. cerevisiae*, which is a more distantly related species. No trimeras were observed in *S. cerevisiae* at multiple time points, during exposure to several FLC concentrations. This suggests that the delay in bud emergence relative to nuclear and spindle cycling and the formation of trimeras is a common response of the CUG clade, as well as the distantly related pathogen *C. glabrata*, to FLC exposure.

### Trimeras Form During Exposure to FLC Within the Host

**Figure 8:**
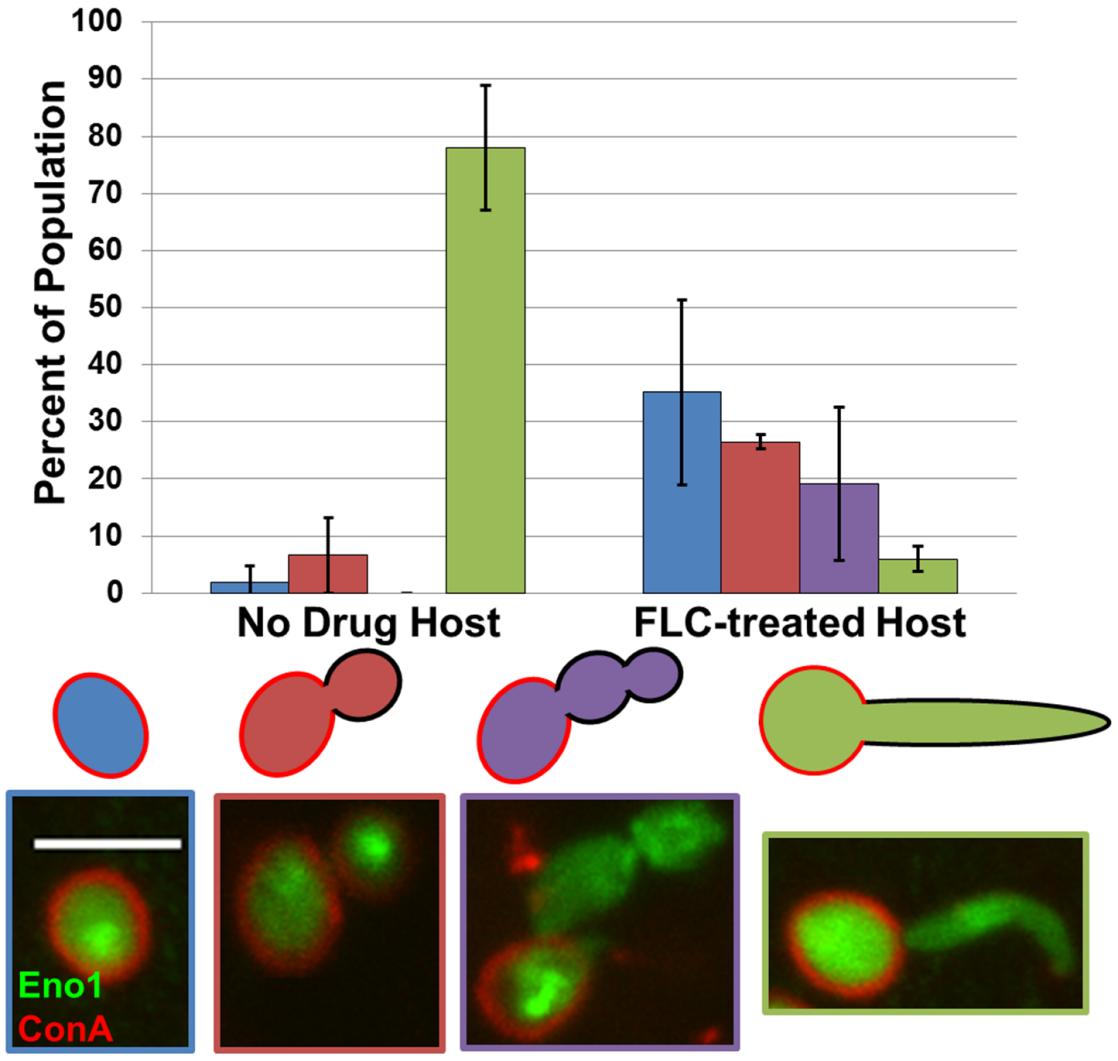
Trimeras form *in vivo*. Percent of ConA-Texas Red/Eno1-GFP cells showing unbudded (blue), budded (red), trimera-like (purple), and hyphal (green) phenotypes within untreated (left, *n* = 195) and FLC-treated (right, *n* = 309) mouse host 48 h after injection. Error bars are 1 standard deviation. Scale bar, 5 µm.

Approximately 50% of FLC^R^ strains from lab and clinical isolates are aneuploid [8]. Based on the data presented above, trimera cells represent a possible avenue for, as well as a cellular indicator of, the appearance of aneuploidy in *C. albicans* and other related species. A critical question is whether trimeras are an intermediate in the formation of aneuploid cells *in vivo* and thus might represent a clinically important step in the development of drug resistance. To address this question, we exploited a mouse model of candidiasis that enables imaging of live *C. albicans* cells within mouse ear tissue (Figure 8) [25]. Mice were inoculated with cells expressing Eno1-GFP, which localizes diffusely in the cytoplasm and is enriched within the nucleus [26], and stained with Texas Red conjugated to Concanavalin A, which stains the cell wall of the originally inoculated cells; cells lacking ConA stained walls were progeny produced *in vivo*. FLC (250 µg/ml) was added to the drinking water for the “FLC” mice. Confocal microscopy of the intact ear of anaesthetized mice was used to detect fluorescently labeled yeast cells as they grew within the ear tissue. After 48 h in the host, the majority of *C. albicans* cells within “no drug” mice had produced hyphae several microns in length (Figure 8). In contrast, after 48 h of growth within FLC mice, *C. albicans* cells were primarily round or elliptical and the trimera-like morphology was evident (19.1% of cells, *n* = 309). No trimera-like cells were detected in the “no FLC” mice (*n* = 195). Thus, FLC exposure leads to trimera formation of *C. albicans* cells *in vivo* as well as *in vitro*.

### Trimera Formation Is Not a General Stress Response

**Figure 9:**
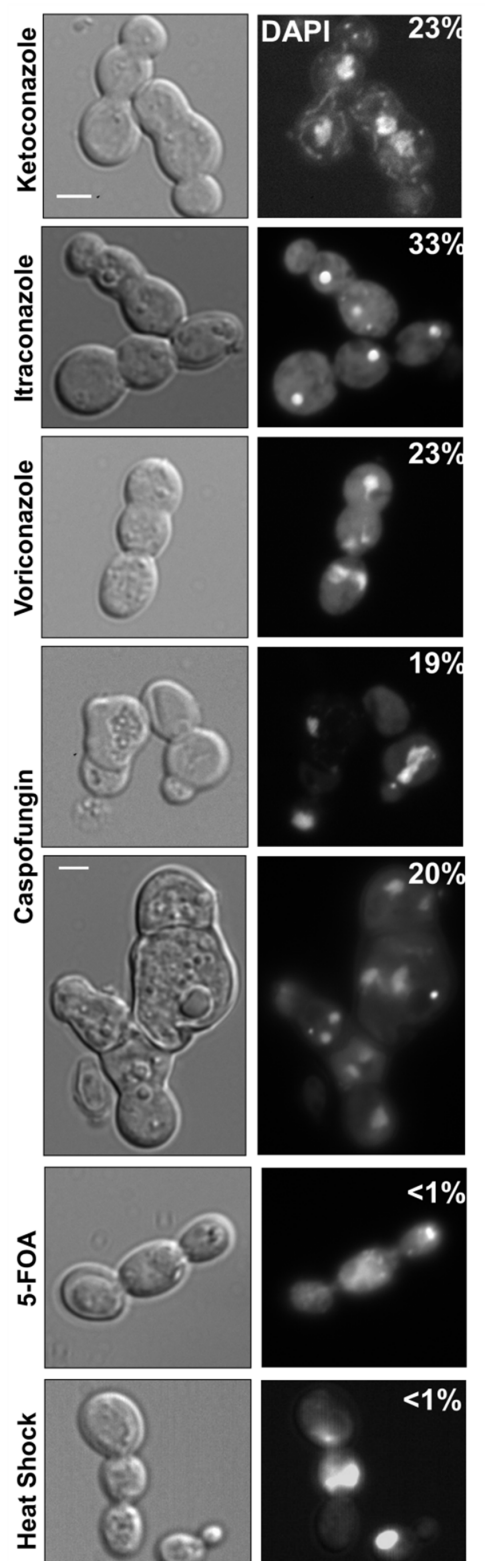
Trimera formation is not a general stress response. Cells exposed to triazoles ketoconazole, voriconazole, and itraconazole formed trimeras at frequencies similar to FLC. Cells that were exposed to caspofungin, an echinocandidn, also produced many trimera-like and multimera-like cells (upper and lower panels). Exposure to toxin 5-FOA as well as heat shock did not result in a significant number of trimeras. No trimeras were detectable following exposure to 2-DOG (unpublished data). Percentages in upper right corner of DAPI image denote frequency of trimera formation in 300–400 cells.

We also asked if exposure to different stress conditions would result in trimera formation. Importantly, exposure to other triazoles gave rise to trimeras (Figure 9). In contrast, exposure to other stresses like 5-fluoroorotic acid (5-FOA) or heat shock caused very little trimera formation (<1%, Figure 9) and no trimeras were detected in 2-deoxygalactose (2-DOG) (unpublished data). These data indicate that trimera formation is not a general stress response of *C. albicans* cells. Interestingly, caspofungin exposure also yielded many cells with defects in cytokinesis resulting in trimera-like and multimera-like morphology (Figure 9), although the mechanisms that gave rise to them remain to be determined.

## Discussion

**Figure 10:**
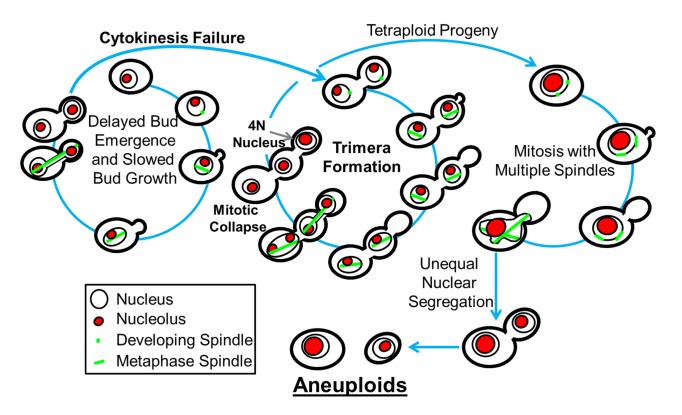
A model for aneuploid formation in *C. albicans* cells exposed to FLC. Proposed model for aneuploid formation in *C. albicans* cells exposed to FLC. Nuclear membrane, thin black line; nucleolus, red; spindle, green.

*C. albicans* FLC^R^ cells are often aneuploid [8]. Here, flow cytometry, time course, and time-lapse fluorescence microscopy together revealed that FLC exposure results in cell cycle delays that ultimately yield a significant subpopulation of aneuploid cells via an ordered series of events including trimeras, tetraploids, and then aneuploids. The series of cell cycle changes is illustrated in Figure 10: first, delayed bud emergence and bud growth relative to nuclear cycling and spindle formation; second, failed cytokinesis; third, budding despite cytokinesis failure to produce trimeras; fourth, tetraploid/dikaryon formation by mitotic collapse; and fifth, aneuploid formation via unequal segregation of tetraploid nuclei with multiple spindles. Importantly, trimeras were viable both in the absence and presence of FLC. Furthermore, trimeras appeared in FLC-exposed CUG clade species as well as in *C. glabrata* but not *S. cerevisiae* and thus are not unique to *C. albicans* and may be a property of pathogenic fungal species. Finally, FLC exposure yields trimeras *in vivo*, and thus they are not an artifact of *in vitro* conditions. Accordingly, we posit that trimeras are an indicator of cell cycle defects and prognosticators of high levels of aneuploidy in the population. Finally, this work provides an explanation for the previous observations that many FLC-exposed and FLC^R^ isolates of *C. albicans* are aneuploid [8].

The mechanism of FLC action is inhibition of ergosterol biosynthesis, which is fungistatic rather than fungicidal. Importantly, the effects of FLC on cell division are not immediate—cells continue to divide for several hours after drug exposure. This suggests that the delay in bud emergence and failure of cytokinesis could be due to gradual depletion of ergosterol and/or accumulation of 14α-Methyl-ergosta 8,24(28)-dien-3,6-diol, the toxic sterol produced by Erg3p when Erg11p is inhibited [27],[28]. Consistent with this, trimeras formed during exposure to several different triazole antifungals (Figure 9). FLC clearly has some effect on membrane dynamics, as in preliminary analysis using FM4-64, a lipophilic styryl dye that intercalates into the plasma membrane and is taken into cells by endocytosis, we found that FLC affected endocytosis, suggesting altered vesicle dynamics and membrane fluidity (R.P., unpublished data). We hypothesize that these effects, in turn, may alter the dynamics of cytokinesis as well as bud growth. Taken together, these results suggest that defective ergosterol biosynthesis is one mechanism that can cause trimera formation, although other mechanisms are also possible.

We previously found that stresses, including exposure to FLC, result in elevated levels of loss of heterozygosity (LOH) [29], which can arise via chromosome missegregation and/or via recombination events. We do not know if trimera formation directly causes such elevated levels of recombination. Recombination is required for the formation of regional and arm LOH events as well as for aneuploidies such as isochromosome 5L (i5L), which confers FLC^R^ in a number of different *C. albicans* isolates [13]. Work in *S. cerevisiae* suggests that the stress of carrying an aneuploid chromosome is sufficient to increase levels of other genome changes [30]. The degree to which *C. albicans* senses aneuploidy as a stress that elevates the levels of other genome changes remains to be determined.

Another possibility is that FLC behaves as a direct mutagen. Some azoles (e.g., nocodazole) cause defects in microtubule dynamics, but FLC had no obvious direct effect on spindle assembly or elongation. Furthermore, a FLC^R^ isolate did not stop dividing in FLC and did not form trimeras (Figure S3). Thus, we hypothesize that FLC directly interferes with cell membrane integrity and that this, in turn, affects other cell cycle processes indirectly. Interestingly, FLC has been reported to induce chromosome breaks and aneuploidy in mouse bone-marrow cells and human lymphocytes [31], indicating that FLC exposure causes aberrant cell cycle events in mammalian cells as well as in *C. albicans*.

Delayed bud emergence was the first obvious defect in FLC-exposed cells. Interestingly, *C. albicans* hyphae undergo hyphal evagination that precedes the initiation of nuclear/spindle cycles at START [16]. This inherent ability for wall growth without initiating the nuclear/spindle cycles normally is not seen in the model yeast *S. cerevisiae* [19]. While the presence of trimeras in *C. albicans*, but not in *S. cerevisiae* (Figure S5), may be a consequence of a difference in the flexibility of cell cycle coordination between these two distantly related organisms, the formation of trimeras in species that do not form true hyphae (including *C. glabrata*, *C. parapsilosis*, *C. lusitaniae*, most isolates of *C. tropicalis*) (Figure S5) suggests that the ability to form hyphae is not linked directly to trimera formation. Furthermore, trimeras formed in mutants that are defective for filamentous growth (strains lacking either Ume6 or lacking both Efg1 and Cph1), indicating that trimera formation requires neither true hyphae nor the cell cycle changes associated with hyphal growth (Figure S6). Furthermore, trimeras formed in mutants lacking Kar3 (a motor protein necessary for nuclear fusion during mating) (Figure S6) as well as in strains that were *MTL* homozygous and/or haploid or tetraploid, suggesting that mating and karyogamy that occurs during mating are not required for trimera formation and that initial cell ploidy also is not a factor.

Intriguingly, caspofungin exposure resulted in many connected cells and the production of trimera-like and multimera-like cells (Figure 9). Caspofungin and other echinocandins perturb ß-glucan synthesis and cell wall integrity. In an earlier study, caspofungin also caused cytokinesis defects in *S. cerevisiae* but not *C. albicans* [32]. However, the strains, growth, and drug exposure conditions here clearly detect cytokinesis failures in a majority of the caspofungin-exposed cells (Figure 9). Whether such cells also form tetraploids and aneuploids remains to be determined. Nonetheless, it appears that defects in wall integrity and/or cytokinesis failure are mechanisms that promote the formation of trimera and multimera cells.

Trimeras provide a distinctive indicator of cell cycle perturbation as they arise through failed cytokinesis between a mother-bud pair followed by evagination of only one rather than two granddaughter buds. Why this happens is not clear. One possibility is that a signal from the first granddaughter is conveyed to both its mother and grandmother, satisfying some yet-to-be characterized bud emergence checkpoint and obviating the initiation of a second bud. However, in *S. cerevisiae* where cell cycle has been examined extensively, a checkpoint that specifically monitors bud emergence or cytokinesis has not been identified and cells with cytokinetic defects are capable of forming two daughter buds [33]. Furthermore, in *C. albicans* the Swe1 morphogenesis checkpoint does not appear to play a role in this process (M.B., unpublished data). Thus, some cell cycle responses to FLC must be regulated differently for *S. cerevisiae* relative to *C. albicans* and other CUG clade members. An alternative, and not mutually exclusive, possibility is that membrane and/or cell wall growth potential is limited by either the low levels of ergosterol in the plasma membrane or by high levels of the toxic sterol intermediate produced by Erg3p.

Trimeras provide a new route to produce tetraploids. In the lab, *C. albicans* tetraploids can form via parasexual mating between two diploids [2]–[4]. Here we found that mitotic collapse within trimeras is a clear alternative mechanism for the formation of viable *C. albicans* tetraploids. Of note, mitotic collapse involves a single nucleus that fails to complete mitosis or re-fusion of two daughter nuclei following mitosis (Figure S4), rather than the fusion of two distinct nuclei that occurs during mating in *S. cerevisiae*. The partial reduction of trimera formation in *kar3* karyogamy mutants could reflect the observation that some, but not all, trimeras undergo a nuclear fusion event that is dependent on Kar3 (Figure S6). We suggest that mitotic collapse may fuel the formation of homozygous autodiploids from haploids as well [7]. Mitotic collapse occurs in ∼75% of trimeras and may be due to failure of the pre-anaphase spindle to align with the bud neck and subsequent spindle elongation within a single cell compartment. Alternatively, cross-talk between spindle disassembly signals in one nucleus could cause early disassembly of the spindle in the other nucleus. This hypothesis is consistent with the observation that spindle disassembly is not affected by FLC exposure (Figure 3B). Regardless of the mechanism, tetraploids become a significant proportion of the population by 8–12 h of FLC exposure.

In addition, we detected multimeras, the very large cells with high DNA content and multiple nucleoli (Figure 1 and Figure 2). We suggest that these extremely large cells are primarily the descendants of trimera cells that continued to undergo aberrant cell cycles. Specifically, we hypothesize that these cells continued to have defects in cytokinesis and bud emergence and continued to undergo DNA replication and mitotic spindle cycles despite the lack of cytokinesis. The expected result would be an increasing imbalance in the number of nuclei relative to the number of cell compartments (Figure 2). Importantly, these large cells, like trimeras, are not necessarily “dead ends” in the culture: they occasionally formed buds that underwent cytokinesis, cell separation, and production of a large but morphologically normal progeny cell that continued to divide (Figure 2 and unpublished data). Extremely large, multinucleate cells that provide a temporary growth advantage are not limited to *C. albicans* or to ascomycete fungi. For example, *Cryptococcus neoformans*, a basidiomycete pathogen of humans forms large “Titan” cells with high ploidy in lung tissue [34],[35] and these cells may confer protection against immune cells. Titan cells also give rise to smaller progeny cells that remain infective [34]. Furthermore, as in *C. albicans*, aneuploidy has been associated with drug resistance in some *C. neoformans* isolates [35], although the degree to which Titan cell progeny are aneuploid remains to be determined.

Aneuploidy is a common feature of mammalian tumors. The mechanism of aneuploid formation in precancerous cells resembles that of aneuploid formation in *C. albicans*: a tetraploid intermediate undergoes mitosis with an excess of spindle components. Human cells can become tetraploid via a failure of cytokinesis that yields a binucleate cell [24],[36]–[38] or by telomere-induced endoreduplication [39],[40]. In the cell cycles following cytokinesis failure, nuclei are associated with extra spindle components that form multipolar intermediates [24]. Mitoses involving extra spindle components increases the level of chromosomal instability due to the formation of merotelic attachments, in which a single sister chromatid attaches to both spindle poles, resulting in lagging chromosomes [24]. Although the general features of tetraploid formation are similar in tumor and *C. albicans* cells, *C. albicans* cells are unlikely to form merotelic attachments, because they usually have a single microtubule attachment site per sister chromatid [41],[42]. Therefore, unequal chromosome segregation in *C. albicans* is most likely mediated by sister chromatids that attach to the same, rather than the opposite, spindle poles and/or by failure of one of the spindles to elongate across the bud neck (type II segregation, Figure 6A).

In general, aneuploidy is considered to be detrimental and to incur a high fitness cost [30],[43]. Yet aneuploidy arises during the normal differentiation of normal mammalian tissues. For example, a significant subpopulation of human neurons and liver cells are aneuploid [44]–[46]. Similarly, hepatocytes undergo an interesting process termed the “ploidy conveyor,” in which cells undergo unusual mitoses to produce ploidy shifts [46]. *In vivo*, the variability generated by the ploidy conveyor contributes to regeneration of liver tissue after toxin-induced damage [47]. Thus, aneuploidy and ploidy shifts may provide selective advantages under certain selective conditions.

In eukaryotic microorganisms, aneuploidies can confer new phenotypes, such as the ability to survive in specific stresses [13],[48], including antifungal drugs [49]. Furthermore, in a number of cases, the fitness cost associated with aneuploidies that confer FLC^R^ in *C. albicans* is low, both in the presence and in the absence of the drug stress [14],[50]. In addition, aneuploidy may provide a transient solution for cells exposed to stressful growth conditions [51]. The high frequency of trimera formation and the large proportion of trimeras that go on to form tetraploids and aneuploids provides a plausible explanation for how drug-resistant aneuploids arise very frequently within drug-exposed *C. albicans* cultures. Accordingly, we propose that FLC induces the cascade of events that leads to frequent tetraploid and aneuploid formation and that a subset of these aneuploid progeny has a selective advantage in the presence of FLC.

Questions remain as to how *C. albicans* is able to alter cell cycle progression such that nuclear cycling initiates prior to bud emergence, as well as how it is able to tolerate such dramatic changes to cell morphology and genetic content. This phenotypic and genetic plasticity is fascinating as a model for other highly plastic cells (e.g., highly aneuploid cancer cells) and also poses major challenges in the clinic. Building on this work, future studies may be able to identify novel drugs and/or drug targets that will aid in the development of more effective treatments to inhibit the ability of *C. albicans* to develop resistance to existing antifungal drugs.

## Materials and Methods

This study was carried out in strict accordance with the recommendations in the Guide for the Care and Use of Laboratory Animals as defined by the National Institutes of Health (Animal Welfare Assurance Number A329201). Animal protocols were reviewed and approved by the University Committee on Animal Resources (UCAR) of the University of Rochester. All animals were housed in an AAALAC-accredited research animal facility.

### Growth Conditions

Strains used in this study are listed in Table S1. Cells were grown to log phase in liquid YPAD (Yeast Peptone Dextrose plus 40 mg/L Adenine; Sigma) [52] media shaken at 25°C in a 125 ml Erlenmeyer flask. For FLC-exposed cultures, FLC was diluted in water and added to a final concentration that was 8–10× their MIC. For cultures exposed to ketoconazole, itraconazole, voriconazole, and caspofungin, cells were grown for 6–10 h in the presence of 1 µg/ml drug (diluted in methanol, chloroform, DMSO, or water, respectively). For cultures exposed to 5-FOA and 2-DOG, drugs were added to a final concentration of 1 µg/ml diluted in water. Cells were maintained in log phase for time course experiments through dilution into fresh media. For mutant strains, MIC was first determined using Etest strips (bioMérieux) and cultures were exposed at 10× MIC (as was the case for wild-type cells).

For heat shock conditions, cells were pregrown in liquid YPAD to log phase, collected by centrifugation, and resuspended in 0.5 ml of preheated (55°C) YPAD medium for a total of 60 s. Cells were then diluted in 10 ml volume of fresh YPAD (25°C), and then grown at 25°C with shaking in YPAD or SD (Synthetic Dextrose medium) [52]. Samples were analyzed by microscopy at t = 0, 4, 6, and 24 h. The cells in Figure 9 are from SD medium at 24 h.

### Flow Cytometry Preparation and Analysis

Mid-log phase cells were harvested, washed, and fixed with 95% ethanol. Cells were then washed and resuspended in 50∶50 TE (50 mM Tris pH 8∶50 mM EDTA). Cells were then treated with 1 mg/ml RNAse A, followed by 5 mg/ml Proteinase K. Cells were washed with 50∶50 TE and resuspended in SybrGreen (1∶85 dilution in 50∶50 TE) incubated overnight at 4°C. Stained cells were collected and resuspended in 50∶50 TE. Data from 25,000 cells per time point were collected using a FACScalibur. Whole genome ploidy was estimated by fitting DNA content data with a multi-Gaussian cell cycle model, and percent of population of each ploidy was calculated using the mean ±3 standard deviations. Ploidy values were calculated by comparing the ratio of peak locations in experimental samples to those of diploid and tetraploid controls.

### Cell Micromanipulation and Analysis

Cells were collected after 12 h of FLC treatment and washed with water. A single stripe of cells was spread onto a thinly poured YPAD plate with or without FLC. Micromanipulation of cells was performed using a Nikon Eclipse E400 microscope equipped with a Nikon 10×, 0.25 NA objective and a tetrad-dissecting apparatus. Colony formation was then scored after plates were incubated for 48 h at 25°C. For flow cytometry of trimeras and trimera progeny, colonies were picked into 250 µl liquid YPAD media in a 96-well block and shaken for 6 h at 25°C. The experiment was repeated on 5 different days using five independent starting cultures.

### FUN1 Staining

FUN1 stain was added to a log phase culture (with or without FLC) to a final concentration of 0.6 µM and shaken, in the dark, for 1 h at 25°C prior to imaging. Cells were washed with water and resuspended in synthetic complete medium containing 2% dextrose (SDC) prior to imaging.

### Time-Lapse Imaging

Time-lapse imaging was performed using microfluidic devices fabricated at the Hervey Krueger Center of Nanotechnology at the Hebrew University of Jerusalem. Briefly, molds were fabricated by photolithography of SU8 photoresist on silicon wafers. Chambers were replica molded in PDMS and bonded to a glass coverslip using oxygen plasma bonding. The chamber was connected to a BD 1 ml syringe via Micro Bore PVC (0.010″ ID) tubing. Chambers were treated with 1 mg/ml conconavalin A for 20 min prior to addition of cells. Cells were added to the chambers and allowed to adhere for 20 min. During imaging, a Chemyx Fusion 200 syringe pump pushed SDC (±10 µg/ml FLC) through the chamber at a rate of 10 µl/h. Imaging of cells was performed on an Olympus DeltaVision Microscope, equipped with an Olympus UPlanSApo 100×, 1.4 NA oil objective and a CoolSNAP ES2-ICX285 camera run by softworX software. Five stack z-series with 500 nm steps were taken at 5 min intervals over a period of approximately 16–20 h.

### Time Course Imaging

Cells in time course experiments were washed and resuspended in SDC prior to being imaged on glass slides with glass coverslips. Experiments involving FUN1, DAPI, and Nop1-GFP were imaged on a Nikon Eclipse E600 microscope equipped with a Nikon 100×, 1.4 NA objective and a Photometrics CoolSnap HQ camera run by Metamorph software. Images were taken using 11 stack z-series with a 350 nm step size. The time course imaging of YJB12626 (Hhf1-GFP) was performed on an Olympus DeltaVision Microscope, equipped with an Olympus UPlanSApo 100×, 1.4 NA oil objective and a CoolSNAP ES2-ICX285 camera run by softworX software. Images were taken in a 21-step z-series with a 200 nm step size. Mutants and cells exposed to drugs other than FLC were imaged on a Nikon Eclipse E600 microscope equipped with a Nikon 100×, 1.4 NA objective and a Clara Interline CCD camera, or for heat shock experiments, a Neo 5.5 sCMOS camera (Andor, Belfast, Ireland).

### Image Analysis

Image analysis of time course experiments involving strains YJB8172 (Nop1-GFP) and YJB12626 (Hhf2-GFP) measuring cell size and or fluorescence intensity was done using a Matlab script developed by Jordan Hashemi and Benjamin Harrison (manuscript in review). Scoring of FUN1 stained cells and quantification of time lapse images was done manually using Fiji software.

### Staining

Cells were isolated from a log phase culture, washed with water, and resuspended in 1× PBS buffer. Stock DAPI solution (10 µg/ml, Sigma Aldrich, Inc.) was added to the cell suspension to a final concentration of 3 µM. Cells were incubated in the dark at room temperature for 5 min. Cells were then washed with water and resuspended in water for imaging.

### Animal Treatment and Housing

*In vivo* imaging experiments were performed similar to those previously described [25]. This study was carried out in strict accordance with the recommendations in the Guide for the Care and Use of Laboratory Animals as defined by the National Institutes of Health (Animal Welfare Assurance Number A329201). Animal protocols were reviewed and approved by the University Committee on Animal Resources (UCAR) of the University of Rochester (this is the University of Rochester's Institutional Animal Care and Use Committee). All animals were housed in an AAALAC-accredited research animal facility. All experimental procedures were approved by the University of Rochester University Committee on Animal Resources (IACUC). Female DBA2/N mice (6–8 wk old) were purchased from the Frederick National Laboratory for Cancer Research (NCI, Frederick, MD). Animals were housed in the University of Rochester Medical Center vivarium and allowed food and water *ad libitum*. For at least 1 wk prior to each experiment, the animals were fed a chlorophyll-free mouse chow to minimize autofluorescence [53]. Animals were treated with FLC by providing them with drinking water containing 0.25 mg/ml FLC starting 3 d prior to inoculation and continuing throughout the experiment. Prior to inoculation, mice were anesthetized with ketamine/xylazine and the hair on the ears was removed by chemical depilation. A 10 µL volume of the *C. albicans* inoculum was then injected intradermally using an insulin syringe. A set of control experiments was performed in which one ear was injected with conA-labeled yeast and the opposite ear was injected with unlabeled yeast. No difference was seen in the progress of infection with or without conA labeling. Thus, subsequent experiments were performed with conA-labeled inocula only. For imaging, mice were anesthetized 43–48 h after inoculation with ketamine/xylazine and placed on the microscope stage in a heated chamber. The ear was immobilized and held flat by trapping it between coverslips.

### *In Vivo* Inoculum Preparation

Cells for the inoculum were grown overnight at 30°C to stationary phase after which they were washed three times in sterile PBS. Cells were then labeled with Texas-Red conjugated Concanavalin A (conA, Invitrogen) at 100 µg/ml for 15 min at room temperature, after which they were washed again, counted in a hemocytometer, and adjusted to a density of 1×10^8^ CFU/ml in sterile PBS.

### *In Vivo* Imaging

Images were acquired using an Olympus FV1000 scanning laser confocal microscope at the University of Rochester Medical Center Microscopy Core facility with a 60× long-working distance objective lens (NA 0.7) with excitation wavelengths of 488 and 559 nm and emission wavelengths of 510 and 603 nm to separate the GFP and Texas Red signals. Images were acquired as a z-stack (1.24 um/step) through the area of infection. The images presented in Figure 8 are maximum-intensity z projection images.

## Supporting Information

Figure S1 **Significant ploidy changes occur within 12 h of FLC exposure.** Flow cytometry profiles of cultures grown in the absence (no drug, top two rows) or presence (+FLC, bottom two rows) of FLC for times indicated (red lines). Gaussian curve fitting (see Materials and Methods) produced ploidy estimates based on diploid (green) and tetraploid (blue) control strains with raw data shown below.
(TIF)

Figure S2 **Trimeras have continuous cytoplasm.** Nucleoli (Nop1, red) and spindles (Tub1, green) move through the bud necks of a trimera.
(TIF)

Figure S3 **FLC^R^ strain does not form trimeras.** The FLC^R^ clinical isolate FH5 (MIC = >64 µg/ml) maintained normal cell morphology in the absence (no drug) and presence of FLC (t_FLC_ = 48). Cells within white boxes were enlarged (bottom row). Scale bars, 5 µm.
(TIF)

Figure S4 **Nuclei re-fuse or fail to separate during mitotic collapse.** Nuclear envelopes during mitotic collapse events imaged using time-lapse microscopy detected with nuclear pore marker Nup49-GFP showed two types of “collapse”: either sister nuclei completed separation and then subsequently re-fused (42%; top two rows) or failed to separate at all (58%; bottom two rows). Total number of cells analyzed was 12. Numbers are time (min) from initial FLC exposure. Arrows denote nuclei that underwent mitotic collapse. Scale bar, 5 µm.
(TIF)

Figure S5 **Non-** ***albicans*** **species exhibit trimera formation.** DIC and fluorescence images of cells from non-*albicans* yeast species stained with DAPI in the absence (no drug, left) and presence (+FLC, right) of FLC. CUG clade members (*C. dubliniensis*, *C. tropicalis*, *C. parapsilosis*, and *C. lusitaniae*) exhibit the trimera phenotype (black arrows in DIC image) and appear to be multinucleate. *C. glabrata*, which is a pathogenic yeast but not a member of the *CUG* clade, also forms trimera-like structures in FLC. We note that the third bud often formed on the mother rather than on the daughter, and we speculate that is due to the different bud-site selection pattern in haploid *C. glabrata* relative to *C. albicans*. *S. cerevisiae*, a nonpathogenic non-CUG clade yeast, did not form trimeras in the absence or presence of FLC. Scale bar, 5 µm.
(TIF)

Figure S6 **Hyphal formation and karyogamy are not prerequisites for trimera formation.** *C. albicans* mutants lacking Ume6 or Cph1 and Efg1 have defects in filamentous growth but when exposed to FLC, they form trimeras (13% and 35% trimeras, respectively; right panels), whereas no trimeras were observed in no drug controls (left panels). A mutant defective in nuclear fusion (lacking Kar3) also formed trimeras at moderate frequencies (6%), possibly because they grow slowly. Mutant genotypes are listed in Table S1.
(TIF)

Movie S1 **Large, multinucleolar cell expressing Nop1-GFP (green).**
(AVI)

Movie S2 **Cell cycle in a no drug control cell with Tub1-GFP (green) and Nop1-RFP (red).**
(AVI)

Movie S3 **Uncoupled nuclear/spindle and bud growth cycles in a cell expressing Tub1-GFP (green) and Nop1-RFP (red).**
(AVI)

Movie S4 **Trimera formation and putative tetraploid cell formation in a cell expressing Nop1-GFP.**
(AVI)

Movie S5 **Trimera formation followed by dikaryon formation in a cell expressing Tub1-GFP (green) and Nop1-RFP (red).**
(AVI)

Movie S6 **Trimera formation followed by mitotic collapse of nucleus (bottom) in a cell expressing Tub1-GFP (green) and Nop1-RFP (red).**
(AVI)

Movie S7 **Tetraploid cell with two spindles that exhibits type I segregation.**
(AVI)

Movie S8 **Tetraploid cell with two spindles that exhibits type II segregation.**
(AVI)

Table S1 **Strains used in this study.**
(DOCX)

## Abbreviations

DIC: differential interference contrast
FLC: fluconazole
FLC^R^: fluconazole resistant
GFP: green fluorescent protein
RFP: red fluorescent protein
SPB: spindle pole body
