# Supplementary figures and images for: A Tetraploid Intermediate Precedes Aneuploid Formation in Yeasts Exposed to Fluconazole
Source: PLoS Biol. 2014 Mar 18;12(3):e1001815. doi: 10.1371/journal.pbio.1001815 (PMC3958355; doi:10.1371/journal.pbio.1001815)

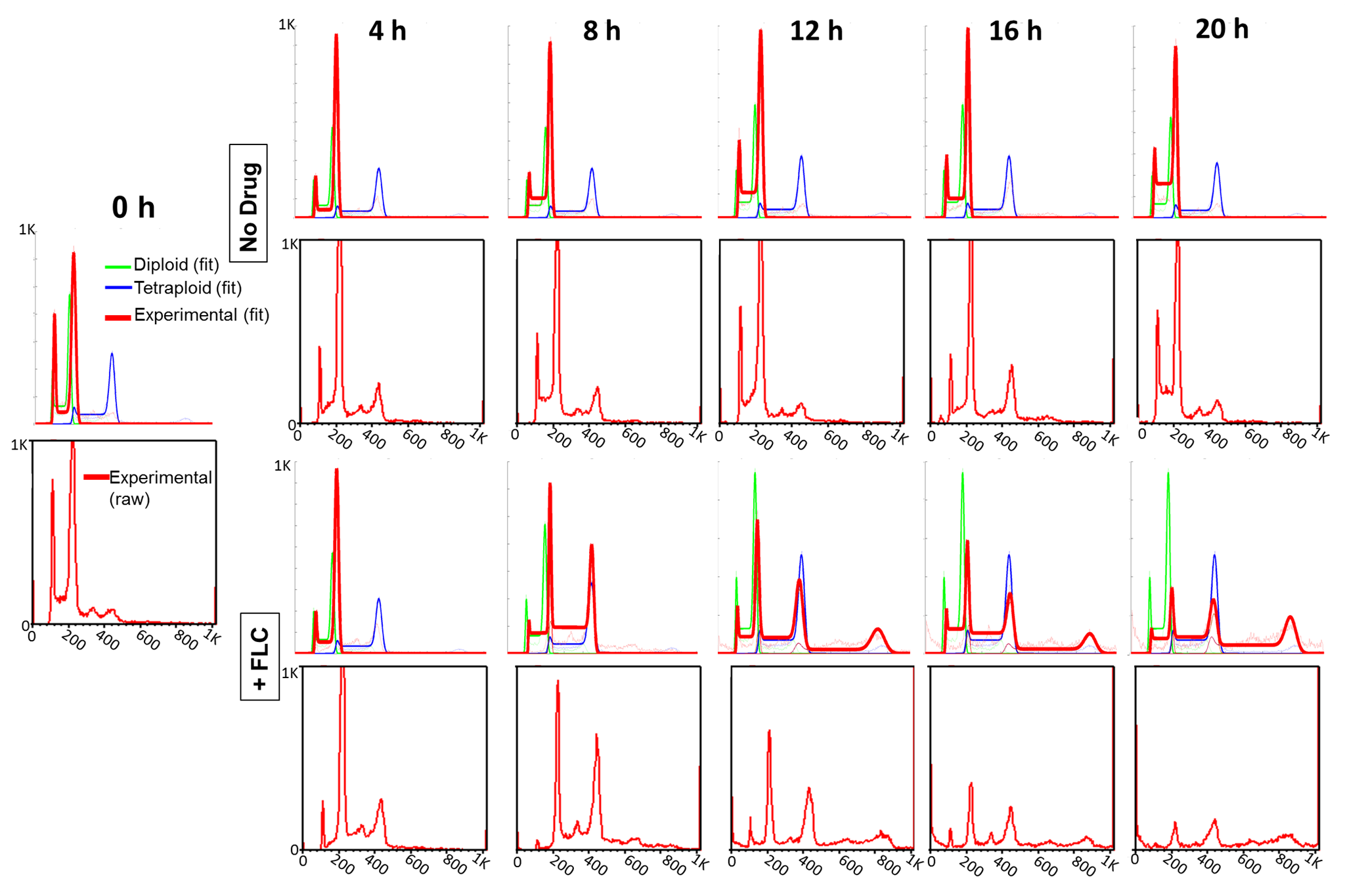

Supplement: Figure S1 — Significant ploidy changes occur within 12 h of FLC exposure. Flow cytometry profiles of cultures grown in the absence (no drug, top two rows) or presence (+FLC, bottom two rows) of FLC for times indicated (red lines). Gaussian curve fitting (see Materials and Methods) produced ploidy estimates based on diploid (green) and tetraploid (blue) control strains with raw data shown below. (TIF) [file pbio.1001815.s001.tif]

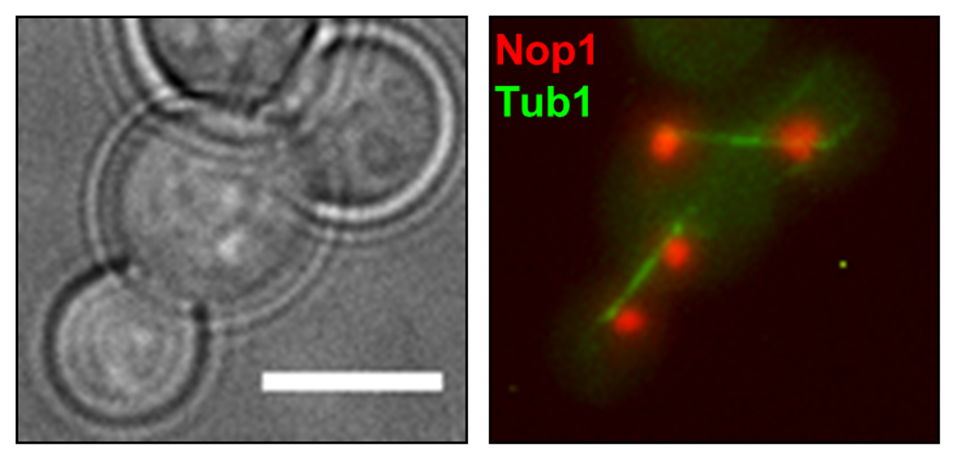

Supplement: Figure S2 — Trimeras have continuous cytoplasm. Nucleoli (Nop1, red) and spindles (Tub1, green) move through the bud necks of a trimera. (TIF) [file pbio.1001815.s002.tif]
