# Supplementary material for: A Tetraploid Intermediate Precedes Aneuploid Formation in Yeasts Exposed to Fluconazole
Source: PLoS Biol. 2014 Mar 18;12(3):e1001815. doi: 10.1371/journal.pbio.1001815 (PMC3958355; doi:10.1371/journal.pbio.1001815)
Supplement: Table S1 — Strains used in this study. (DOCX) [file pbio.1001815.s015.docx]

**Table S1. Strains used in this study**

| **Strain Name** | **Strain name/relevant feature** | **Genotype** | **Source** |
| --- | --- | --- | --- |
| ***C. albicans*** | | | |
| YJB5326 | SC5314 | Wild-type | J. Ernst |
| YJBT676 | CAF3-1 | *ura3∆::imm434 / ura3∆::imm434* | W. Fonzi[1] |
| YJB3731 | BWP17 | CAI-4 *his1::hisG/his1::hisG arg4::hisG/arg4::hisG* | A. Mitchell[2] |
| YJB5957 | *ENO1-GFP* | *ENO1/ENO1::GFP-HIS1* | This study |
| YJB8172 | *NOP1-GFP* | *BWP17 NOP1/NOP1::GFP-HIS1* | This study |
| YJB12626 | *HHF1-GFP TUB4-mCherry* | *BWP17 HHF1/HHF1::GFP-URA3 TUB4/TUB4::mCherry-Nat* | This study |
| YJB12856 | *TUB1-GFP NOP1-RFP* | BWP17 *TUB1/TUB1::GFP-URA3 NOP1/NOP1::RFP-Nat* | This study |
| YJBT677  HLC54 | *efg1∆∆ cph1∆∆* | *ura3∆::imm434 / ura3∆::imm434  cph1∆/cph1∆ ,efg1∆/efg1∆::URA3* | G. Fink[3] |
| YJBT678  HLC69 | *efg1∆∆ cph1∆∆* | *ura3∆::imm434 / ura3∆::imm434  cph1∆/cph1∆ ,efg1∆/efg1∆::URA3* | G. Fink[3] |
| YJBT682 | *ume6∆∆* | *CAF3-1 ume6::hisG/ume6::hisG* | D. Kornitzer |
| YJB7400 | *kar3∆∆* | BWP17 *kar3::UAU1/kar3::URA3* | D. Davis |
| YJB7918 | *kar3∆∆ NOP1-GFP* | *BWP17* *kar3::UAU1/kar3::URA3 NOP1/NOP1::GFP-HIS1* | This study |
| YJB10191  RBY18 | tetraploid | *CAI4 MTLa/MTLα1Δ/α2Δ MTLa∆::URA3/MTLα GAL1/GAL1 gal1Δ::his1/gal1Δ::his1 ADE2/ADE1/ade2::hisG/ade2::hisG;* | R. Bennett[4] |
| YJB12804 | Haploid IV | *MTLα his4 galΔ* | M. Hickman[5] |
| ***Other species*** |  |  |  |
| YJB2491 | YEF473 | *Mata/α ura3-52/ura3-52, leu2-Δ2/leu2-Δ2, his3-Δ200/his3-Δ200, trp1-Δ63/ trp1-Δ63, lys2-801/ lys2-801 S. cerevisiae* | J. Pringle |
| YJBT657 | BG14 | *Wild-type C. glabrata* | B. Cormack |
| YJB12512 | Wu284 | *Wild-type C. dubliniensis* | D. Sullivan |
| YJB4170 | 660 | *Wild-type C. tropicalis* | P.T.Magee |
| YJB12108 | ATCC22109 | *Wild-type C. parapsilosis* | C. Bendel |
| YJB10261 | Can133 | *Wild-type C. lusitaniae* | M. Lorenz |

**Table References**

1. Fonzi WA, Irwin MY (1993) Isogenic strain construction and gene mapping in Candida albicans. Genetics 134: 717–728.

2. Wilson RB, Davis D, Mitchell AP (1999) Rapid hypothesis testing with Candida albicans through gene disruption with short homology regions. Journal of Bacteriology 181: 1868–1874.

3. Lo HJ, Kohler JR, DiDomenico B, Loebenberg D, Cacciapuoti A, et al. (1997) NonfilamentousC. albicansmutants are avirulent. Cell 90: 939–949.

4. Bennett RJ, Johnson AD (2003) Completion of a parasexual cycle inCandida albicansby induced chromosome loss in tetraploid strains. The EmBO Journal 22: 2505–2515.

5. Hickman MA, Zeng G, Forche A, Hirakawa MP, Abbey D, et al. (2013) The “obligate diploid” Candida albicans forms mating-competent haploids. Nature 494: 55–59. doi:10.1038/nature11865.
